# Health care provider perception of chronic kidney disease: knowledge and behavior among African American patients

**DOI:** 10.1186/1471-2369-15-112

**Published:** 2014-07-10

**Authors:** Abby S Kazley, Emily E Johnson, Kit N Simpson, Kenneth D Chavin, Prabhakar Baliga

**Affiliations:** 1 Department of Health Care Leadership and Management, MUSC, 151 Rutledge Ave, Charleston, SC 29425, USA; 2 Department of Surgery Division of Transplantation, MUSC, 96 Jonathan Lucas Street, Charleston, SC 29425, USA

**Keywords:** Chronic kidney disease, African American, Disparities

## Abstract

**Background:**

Chronic Kidney disease (CKD) is prevalent in the population, but also disproportionately affects African Americans. Disparities in care of chronic kidney disease and transplant for African Americans have also been reported. The purpose of this study is to determine the knowledge and reactions of chronic kidney disease patients regarding their disease, as perceived by nephrologists and clinic nurses in South Carolina.

**Methods:**

Using a qualitative approach, key informant interviews were conducted with nephrologists, and three focus groups were held with nurses who specialize in chronic kidney disease. The results were recorded, transcribed, and analyzed using Qualrus software and the Grounded Theory Method.

**Results:**

Dominant themes in the interviews and focus groups include: reaction to chronic kidney disease, differences in race, patient thoughts on dialysis, patient knowledge of types of treatment available, information availability, compliance to treatment, information source, and thoughts on kidney transplantation. The study found that the majority of clinicians agreed that there is typically a wide range of reactions in patients with chronic kidney disease.

**Conclusions:**

The majority of chronic kidney disease patients remain in denial of their diagnosis and do not want to agree to the necessary treatment to improve their condition. In addition, the clinicians reported that the incidence of chronic kidney disease is highest in the African American population and this population of patients typically gets their information on the disease from peers, others they have known that have had renal failure. We find clinicians report that patients typically do not remain compliant to recommended treatment regimens due to lack of knowledge and feelings of denial and fear, and frequently use religiosity as a coping mechanism. Silent progression and complexity of chronic kidney disease frequently result in many patients lacking essential knowledge and developing poor coping mechanisms to seek appropriate follow-up care and prevent progression and optimize outcome. Health care providers are aware of the barriers but may lack the tools and resources to overcome them.

## Background

Chronic kidney disease (CKD) is a progressive condition that is a significant contributor to mortality and morbidity in the United States. Twenty million people older than twenty years of age, or more than 10% of all people have CKD [1]. CKD can lead to End Stage Renal Disease, and costs related to End Stage Renal Disease have been found to be high, as public and private funds paid more than $40 billion in 2009 with annual treatment costs per patient an average $70,000 [2]. End Stage Renal Disease rates are more than three times higher for African Americans than for Caucasians [2].

It has been established that African Americans have a higher incidence and prevalence of CKD than any other racial and ethnic group. They also require dialysis or transplant at younger ages than any other group. These disparities in incidence and prevalence have been proven to be a function of high levels of CKD risk factors in African Americans, including diabetes, hypertension, and obesity [3-9].

In addition, Nezerue, et al. found that African Americans have lower access to medical treatment and transplant most likely due to social and environmental factors related to CKD including poverty and low income, lack of health insurance, lower education, inner city residence, substance abuse, poor diet, obesity, stress, and cultural factors [9]. Lenz, et al. found similar results in their study of the proportion of CKD patients that meet established clinical care guidelines for treatment and kidney function improvement [10]. Studies recommend that physicians should ideally serve as advocates for this high risk population by leading individualized and structured counseling and educational programs, in order to ensure these high risk populations get the care they need and to reduce the health disparities evident with CKD [9,10]. Physicians must also pay attention to cultural and behavioral factors that play a role in health care and lifestyle decision making in this population. In addition, primary care providers should offer early referrals to nephrologists to ensure patients get the specialty care they need [9,10].

African Americans disproportionally use religion as a coping mechanism. Tanyi and Werner compared African American and Caucasian women with end stage renal disease who were getting treatment at a dialysis clinic [11]. They were compared in feelings of well- being and spirituality strengths and it was found that African American women had stronger feelings of spirituality as demonstrated in feelings of God’s concern for them, God’s help with loneliness, and a sense of fulfillment in their relationship with God. In general, higher levels of spirituality have been associated with more positive illness adjustment and better health outcomes. The authors concluded that combining spirituality elements and focusing on these factors are vital in disease treatment methods, especially amongst African Americans [11].

In a series of focus groups with a large African American population, researchers concluded the need for education on risk factors of renal disease such as diabetes, hypertension and cardiovascular disease and improved communication and accessibility with physicians [12]. The participants did possess general knowledge of kidney function and some behavioral risk factors for CKD and were able to relate that affordability of health care, medicine costs, and lack of insurance were the largest barriers to receiving health care.

Waterman, et al. surveyed African Americans about their attitudes and behaviors regarding early detection of CKD [13]. From their telephone survey research, they found that only 23% of the African Americans surveyed were screened for CKD in the past year and although almost half of the respondents had a risk factor, only 3% reported that CKD was a health concern to them. Survey participants were mostly unaware of the higher risk of African Americans in developing CKD and that there was a test to diagnose the disease. A similar study also reported that survey results found that African Americans were typically unaware that they had a higher risk for CKD [14]. And one more additional survey study conducted with a majority population of African Americans with either diabetes or hypertension found that even patients that had high risk factors for CKD, including diabetes and hypertension, did not perceive high susceptibility of developing the disease and were not concerned with the risk of it [15]. All three sets of these researchers concluded that African Americans typically have poor knowledge about CKD, do not perceive it as an important health issue or concern, and are not getting routine screenings for the condition. They recommended educational efforts aimed at the African American population in order to increase early detection of the disease [13-15].

Lea and Nicholas agreed that intervention programs should be aimed at preventing risk factors for CKD (diabetes, hypertension, obesity) in the African American at risk population [8]. The authors pointed out that education on lifestyle modifications are vital because of increased social, environmental, and genetic factors that this population faces. In addition, improved access to health care and policy changes for health care reimbursement would improve the high CKD prevalence in this population.

Compliance to available treatment options has been found to be an issue in African Americans with CKD. Jean-Baptiste determined the relationship between mental and physical health related quality of life, self- esteem, self-efficacy, and hope with compliance to dialysis treatment among a group of African Americans [16]. She found that high ratings of patient self -efficacy and hope were protective against non-compliance with dialysis treatment which suggests that fostering these constructs in dialysis patients may improve compliance rates.

An article by Brown, et al. discussed the kidney early evaluation program (KEEP) which had the goal to screen individuals with high risk for CKD (individuals with diabetes, hypertension, or obesity) in order to prevent and delay the progression of the disease [7]. By screening high risk individuals, the program was able to focus the screening and medical attention to the population that needed it most and focus valuable medical dollars to preventing development and spread of CKD.

The previously discussed research illustrates the lack of knowledge of CKD risk factors and lack of screening performed in one of the highest risk populations, African Americans. All researchers who have completed studies on the topic of CKD are in agreement that early prevention and education programs should be aimed at high risk populations of African Americans and those with diabetes, hypertension, and obesity and programs need to focus on cultural, behavioral, and societal factors applicable to this group.

Focus group data analysis typically consists of a group with a number of individuals that are homogenous in some manner and generally do not have previous experience with each other. The focus group is brought together for research purposes and consists of a conversation lasting for an hour to two hours, led by one or two moderators and provides qualitative data on a specific research topic [17]. Due to the “group effect” individual members of the focus group will build upon other’s responses to the questions and issues being discussed and will be able to expand on each other’s knowledge, therefore making the responses richer, more elaborate, and thus more valuable to data collection [17-19]. Therefore, the purpose of this study was to determine the knowledge and reactions of CKD patients regarding their disease, as told by a group of nephrologists at the Medical University of South Carolina and a group of dialysis and nephrology nurse clinicians from around the state of South Carolina. African Americans were chosen because of their increased risk and prevalence of CKD in South Carolina, and nephrologists and nurse clinicians were chosen because they have a unique perspective from treating these patients each day.

## Methods

### Setting and subjects

Four community-based nephrologists participated in individual key informant interviews at the Medical University of South Carolina in Charleston. Individual nephrologists who regularly referred patients to the Medical University of South Carolina were personally invited in writing to participate. Interviews involved open ended questions regarding nephrologist feedback and thoughts on patient reactions to a diagnosis of CKD, racial differences in prevalence of CKD, role of patient faith in incidence and treatment of CKD, patient beliefs of causes of CKD, patient knowledge of treatments available for CKD, thoughts on the different types of treatments, patient source of information about the disease and treatment options, and other individual thoughts and opinions related to this condition. Nephrologists were asked to consider their African American patients with CKD because of the prevalence of this population in South Carolina.

Three different focus groups of nurse clinicians were held in Charleston and Columbia, South Carolina. Individuals who worked at practices that regularly referred to the Medical University of South Carolina were invited by written letter to participate in the focus group. They were given a choice of times based on convenience. The three focus groups included dialysis center nurses, clinic nurses from MUSC in Charleston and clinic nurses from Columbia. These included both registered nurses and nurse practitioners. Interviews involved open ended questions regarding nurse feedback and thoughts on patient reactions to a diagnosis of CKD, patient knowledge and opinions of different types of treatment for CKD, rate of patient compliance to treatment and reasons for non-compliance, role of faith and religion in patient’s ability to cope with CKD and treatments, and available information and support for CKD patients. Nurse clinicians were also were asked to consider their African American patients with CKD because of the prevalence of this population in South Carolina.

The study was approved by the Medical University of South Carolina’s Institutional Review Board, and written consent was obtained from all focus group and key informant interview participants prior to the data collection. Furthermore, the data was de-identified to protect the providers’ anonymity. The focus groups and interviews were conducted by two experienced qualitative researchers who were both present for each session.

### Qualitative data analysis

Four separate interviews of community-based nephrologists and three separate focus groups of specialized CKD nurse clinicians were conducted and audiotaped. The interviews were transcribed into word documents and then downloaded to the qualitative coding software, Qualrus. This software program allows a researcher to mark segments of text, video, or picture files with qualitative codes and produce useful summaries of the data. Qualrus offers many valuable tools for the finalized data sets, including a variety of statistical summaries of the codes and also a manner in which to categorize, refine and generalize the different codes. Two different coders, including a health services researcher with more than twenty years’ experience with qualitative research and a graduate student, analyzed the data.

Utilizing Qualrus to qualitatively code the interview data from all clinician files together provided a systematic method of coding. A coding ‘scheme’ was created to identify the main thoughts and ideas presented by the clinicians and this scheme was used consistently and continuously for all data files. The same codes were utilized for each of the four clinician files and once the first two files were completely coded, no additional new codes needed to be created for the third and fourth files. This signifies the notion of saturation of codes, meaning that four clinician interviews were adequate to attain all relevant and necessary data needed for our study since ideas and thoughts were consistent throughout the four data files. At the point of saturation, no new concepts will emerge from reviewing additional data of additional participants [20]. Past research has stated that the codes and code structure can be finalized at the point of saturation [21-23]. Once all files were coded entirely and deemed ‘finalized’, the researcher double checked each file for consistency and accuracy of codes.

The Grounded Theory Method was used for coding the interview data files. In this method, the researcher utilizes an inductive approach to developing a set of codes. The data is reviewed line by line, and a code is assigned as a concept becomes clear. The researcher continues to assign codes in the data and refines them as more concepts appear. Once all data is coded, the researcher compares the text segments to ensure that segments are appropriately coded [20]. In grounded theory method of qualitative analysis, after the data is coded themes can emerge and be classified. Themes have been defined as categories that emerge from participant experiences and provide unifying ideas regarding the research topic [20]. In the current study, the researcher utilized Qualrus to identify and classify the codes into general themes that represent the nephrologist’s thoughts and ideas.

## Results

During the interviews, it was evident that the nephrologists were very aware of their patient’s knowledge, ideas, and beliefs surrounding CKD and its effects and consequences, as the clinicians were able to give detailed information on patient experiences with the condition. Some of the most common topics in the nephrologist and nurse clinician interviews centered around patient reactions to a diagnosis of CKD, differences in race surrounding CKD, patient thoughts on dialysis, patient knowledge of types of treatment available CKD, patient reactions to treatment, patient thoughts and beliefs in God relating to CKD, and patient thoughts on causes of CKD.

### Themes in Nephrologists

Themes from the nephrologist file data are listed below (Table 1), along with the corresponding number of segments that each theme represented in all of the four files:

**Table 1:** Themes in nephrologist data for African American patient perceptions on chronic kidney disease

**Reaction to chronic kidney disease (29 segments)**	**Definition of code**	**Number of occurrences**
Chronic Kidney disease difficult to understand	When patient diagnosed with chronic kidney disease, they do not understand all aspects	7
Denial reaction to chronic kidney disease	When patient diagnosed with chronic kidney disease, they go into denial	6
Shock reaction to chronic kidney disease	When patient diagnosed with chronic kidney disease, they go into shock	6
Restricted in lifestyle due to chronic kidney disease	When patient diagnosed with chronic kidney disease, they realize they will have restricted lifestyle	4
Death is a risk from chronic kidney disease	When patient diagnosed with chronic kidney disease, they realize that death is a risk	3
Fear reaction to chronic kidney disease	When patient diagnosed with chronic kidney disease, they have fear	2
No reaction to chronic kidney disease	When patient diagnosed with chronic kidney disease, they have no reaction	1
**Differences in race (22 segments)**	**Definition of code**	**Number of occurrences**
Af Am have more experiences	African Americans have more experiences with chronic kidney disease	5
Af Am have more chronic kidney disease	African Americans have higher prevalence of chronic kidney disease	4
Af Am see conditions as out of control	African Americans see effects of chronic kidney disease as out of their control	2
Af Am have more diabetes	African Americans have higher prevalence of diabetes	2
Af Am have more high blood pressure	African Americans have higher prevalence of hypertension	2
Af Am ask less questions	African Americans ask less questions of their physicians	2
Caucasians ask more questions	Caucasians ask more questions of their physicians	2
Af Am trust in God	African Americans talk about trusting in God more to physicians	2
Af Am have less anxiety	African Americans have less anxiety relating to kidney disease	1
**Thoughts on dialysis (20 segments)**	**Definition of code**	**Number of occurrences**
Fear of dialysis	Patient has fear of dialysis	17
Dialysis helps chronic kidney disease	Patient thinks that dialysis helps treat chronic kidney disease	3
**Types of treatment (19 segments)**	**Definition of code**	**Number of occurrences**
Dialysis is a treatment for chronic kidney disease	Dialysis is a treatment for chronic kidney disease	7
Transplant is a treatment for chronic kidney disease	Transplant is a treatment for chronic kidney disease	5
Older home remedies as treatments for chronic kidney disease	Older home remedies are treatments for chronic kidney disease	4
Drinking water helps chronic kidney disease	Drinking water is a treatment for chronic kidney disease	2
Healthy diet helps chronic kidney disease	Healthy diet is a treatment for chronic kidney disease	1
**Reaction to treatment (14 segments)**	**Definition of code**	**Number of occurrences**
Patient does not want treatment	Patient does not want chronic kidney disease treatment	12
Patient compliant with treatment	Patient is compliant with chronic kidney disease treatment	2
**Thoughts on God (11 segments)**	**Definition of code**	**Number of occurrences**
God helps situation	God can help the situation	6
God not responsible	God does not help situation but is not responsible for chronic kidney disease	3
God is in control	God does not help or hinder the situation but is in control of outcomes	2
**Causes of kidney disease (11 segments)**	**Definition of code**	**Number of occurrences**
Unsure of causes of chronic kidney disease	Patient is unsure of causes of chronic kidney disease	4
Blood pressure causes chronic kidney disease	Patient believes that hypertension causes chronic kidney disease	2
Diabetes causes chronic kidney disease	Patient believes that diabetes causes chronic kidney disease	2
Genetics causes chronic kidney disease	Patient believes that genetics causes chronic kidney disease	1
Too much protein causes chronic kidney disease	Patient believes that eating too much protein is a cause of chronic kidney disease	1
Poor diet causes chronic kidney disease	Patient believes that poor diet is a cause of chronic kidney disease	1
**Information source (7 segments)**	**Definition of code**	**Number of occurrences**
Experience gave information	Patient got information on chronic kidney disease from personal experiences with family members or career	4
Internet gave information	Patient got information on chronic kidney disease from the Internet	3
**Reasons for treatment non-compliance (7 segments)**	**Definition of code**	**Number of occurrences**
Patient not aware of treatment	Patient is non-compliant with treatment because unaware of what is available	2
Patient does not like treatment	Patient is non-compliant with treatment because they do not like the treatment	2
Handicap reason for non compliance	Patient is non-compliant with treatment because they are handicap	1
Lack of intelligence reason for non compliance	Patient is non-compliant with treatment because lacks basic intelligence for every-day life	1
God will treat	Patient is non-compliant with treatment because believes God will treat	1
**Sign of chronic kidney disease (5 segments)**	**Definition of code**	**Number of occurrences**
No symptoms of chronic kidney disease	Patient has no symptoms for chronic kidney disease	3
Nausea is a sign of chronic kidney disease	Patient has nausea as a symptom of chronic kidney disease	1
Pain is a sign of chronic kidney disease	Patient has pain as a symptom of chronic kidney disease	1
**Chronic kidney disease occurrence (5 segments)**	**Definition of code**	**Number of occurrences**
Af Am have more chronic kidney disease	African Americans have higher prevalence of chronic kidney disease	3
Lower education level have more chronic kidney disease	Lower education population has higher prevalence of chronic kidney disease	2
**Transplant thoughts (3 segments)**	**Definition of code**	**Number of occurrences**
Transplant is magic cure	Patient thinks that transplant is a magic cure for chronic kidney disease	2
Unsure of transplant	Patient is unsure of benefits of transplant in chronic kidney disease	1
**Home dialysis (3 segments)**	**Definition of code**	**Number of occurrences**
Home dialysis intimidating to patients	Patients are intimidated at the thought of home dialysis	3
**Information availability (1 segment)**	**Definition of code**	**Number of occurrences**
Lack of information	Patient does not have access to information	1

Reaction to CKD (29), differences in race (22), thoughts on dialysis (20), types of treatment (19), fear of dialysis (17), reaction to treatment (14), thoughts on God (11), causes of CKD (11), information source (7), reasons for treatment non-compliance (7), signs of CKD (5), occurrence of kidney disease (5), transplant thoughts (3), home dialysis (3), information availability (1)

### Most prevalent themes

#### Reaction to CKD

Each nephrologist in the four interviews spoke broadly about patient reactions to a diagnosis of CKD. Patient reactions range from no reaction to intense fear, shock, and denial. As one nephrologist stated, “I have all kinds of things [reactions]; I have some people that don’t bat an eye lash…” and one physician also volunteered “…they don’t understand or they’re shocked, they don’t want to deal with it", signifying the range of emotions and reactions seen from CKD patients.

Nephrologists stated multiple times that CKD is a very difficult disease to understand and one of the reasons that patients have a hard time with a diagnosis is that there are many complex facets of the disease. “CKD, as you know, is very strange for the lay person to understand… nobody knows what a nephrologist is and they don’t know what creatinine is and they really don’t know why they were sent [to the physician office]". A different nephrologist echoed these words, “…the bulk of them don’t know what it is, haven’t heard of it, most of them who come in don’t even know why they are there… and is it not a concrete thing they can get their head around sometimes".

Nephrologists also acknowledged that patients with a diagnosis of CKD realize that they will have to face restrictions in their typical and routine lifestyle. Nephrologists reported that some patients are accepting of the restricted lifestyle; however, some have a much more difficult time with having their daily routine activities impacted and altered. In describing this, one physician said, "…so yes, they feel the burden of this disease. I don’t hear them complaining too much, you know, “I can’t do this; I can’t do that, because of the disease”. But yes, they feel the burden of this, and this disease does have an impact on them. They cannot do this, they cannot do that".

After receiving a diagnosis of CKD, nephrologists also recognized that patients realize death is a potential danger from the condition. The nephrologists spoke about the degree of ‘fatalism’ seen in patient questions and worries, and thought that one of the main reasons some of the patients are accepting of the new restricted lifestyle is that they realize the seriousness of their disease.

#### Differences in race

The nephrologists in all four interviews also expressed differences in various racial groups, in multiple aspects relating to CKD. It was reported that African Americans have more prevalence of CKD and also have more previous experience with CKD from friends and family members with the condition. Relating to this, clinicians stated that they saw trends of African Americans asking fewer questions in the physician office, than their Caucasian counterparts, possibly due to their previous experiences and preconceived notions with the disease. One nephrologist explained potential reasoning for this, “in [the] African American population… the people who sort of drag their feet with it, don’t want to talk about it, don’t want to get an access [for dialysis] placed. It’s often because they know someone who has been on dialysis and not done well". Another nephrologist agreed with this idea, “in the African American population this is very common. So almost everybody knows somebody from the church or has a relative that went through this, so a lot have preconceived notions". One nephrologist brought up a positive point of the increased experience with CKD by stating that he has seen that since African Americans have more experience with the disease, they also exhibit less anxiety related to the condition and its effects.

Nephrologists also reported that trends show that the African American population has a higher prevalence of hypertension and diabetes. In addition, when speaking of faith and beliefs, nephrologists conveyed that African Americans demonstrate and reveal more reliance on religion and God to help and heal them. African Americans report more often than their Caucasian counterparts that they feel that their health conditions are out of their control and they have faith and trust in God to ameliorate their condition. One African American patient was quoted as saying, “not that those things [medical intervention] would fix the kidneys, but that God has the final say and that whatever will happen, will happen because we have no control, God has control".

#### Thoughts on dialysis/fear of dialysis

Nephrologists spoke in each interview about patient’s perceived thoughts on dialysis, and the majority of conversation concluded that patients are fearful of dialysis and dislike the procedure. Previous experience, preconceived notions and general knowledge of dialysis causes patients to be fearful of the procedure. A nephrologist explained that she dislikes speaking about dialysis with patients because of the reaction, “…the moment you tell them dialysis, you see tears running down their cheeks”. One nephrologist explained that experience and knowledge gives patients fear, “you have a [dialysis] room with 16 people in it, every six [to] eight months someone dies, disappears or didn’t come in, where is that person? I’m sure they’re wondering; you never hear it much from them, when will it be my turn, when will it be their turn? “Other nephrologists described that it is not only fear that stems from dialysis, but also dislike, as patients simply do not like having to go through the procedure multiple times a week, even though some of them do understand that dialysis is vital for them. “I’ve had people complain, they have been on the machine the past two years/three years, they understand this is what is keeping them alive. But at the same time they hate it. They have told me, “Doc I just hate it, I can’t help it”. Their blood pressure shoots up the moment they come to the dialysis clinic and they are just miserable”.

#### Types of treatment

When nephrologists spoke on their patient’s knowledge of treatment options for CKD, they reported that most patients are mainly knowledgeable of dialysis and kidney transplant. However, some patients also list older home remedies as potential treatments, including taking long naps, drinking special tea, and taking herbal supplements. In addition, a handful of patients maintain that drinking more water and a healthy diet are possible treatments for CKD.

#### Reaction to treatment

Although stated above that patients are eventually somewhat accepting of their new restricted lifestyle they must lead with CKD, once they first receive a diagnosis of CKD, they are not typically enthusiastic or proactive about beginning treatment. The nephrologists reported that the majority of patients bluntly state that they do want to undergo treatment for CKD, verbally refuse medical procedures, and purposely skip medical appointments. One nephrologist admitted, “I spend a lot of time trying to convince them that they are progressing and that we will have to do something about it. We will have to talk about dialysis and transplantation and there are a few that are very proactive and are willing to do that preemptively. I would say the vast majority of them are not".

#### Thoughts on God

Nephrologists spoke openly about their patient’s faith and beliefs in religion and God, as it relates to their health and medical care. They reported that the majority of patients believe that God helps the situation and will help with healing and improving their condition. Patients were quoted as saying that God will take care of them, heal them, and help them. Nephrologists agreed that having faith in a higher being is typically beneficial to improving a patient’s condition and sometimes provides the extra support a patient needs to thrive from medical treatments. However, the negative aspect to this plays a role when a patient does not want to undergo necessary medical treatment because they believe that God will heal them, without medical intervention. One nephrologist supported this idea by repeating statements he has heard from his patients, ““God is going to help me deal with this, or God is going to help me heal this. I’m not going to need dialysis; Jesus is going to take care of it””.

Two other aspects related to faith were discussed in the interviews with nephrologists. It was reported that some patients believe that while God will not help the situation, God is not at all responsible for this disease. The patient has CKD and does not want to commit to the belief that God will help heal them, but also does not blame it on God’s will. In addition, a separate set of patients have the belief that God is in control and while God may not help or hinder medical treatment, whatever will happen, will be because God has the power to control the results.

#### Causes of CKD

When asked of their patient’s beliefs of causes of CKD, the nephrologists admitted that the majority of patients are unsure of possible causes. One nephrologist stated “…when they come to see us on dialysis and we ask them, what caused your kidneys to fail? You always get a blank look". Another nephrologist reported that patient’s typically ask him what they did wrong to cause the disease because they do not know potential causes. Other patients are aware that diabetes, hypertension, genetics, a poor diet, and eating too much protein are causes of CKD.

### Other facets discussed in the focus groups

In addition to the main themes discussed above, nephrologists volunteered other opinions and experiences of their patients, relating to CKD.

Information source-in discussing the source for patient’s information on CKD, clinicians stated that most patients get their knowledge from previous experience with friends or family members. Most of the time, these patients have experienced the negative side effects of the disease and treatments so have negative preconceived notions before their initial medical appointment with a physician. In addition, the nephrologists reported that more affluent and well-educated patients are able to get information from the Internet and these patients typically come to medical appointments prepared with lists of questions which are helpful to clinicians. However, these patients have also read about negative aspects of the disease on the Internet and already have engrained fears of CKD and available treatments.

Reasons for treatment non-compliance-clinicians stated that some patients are not compliant with necessary treatment for their CKD and there were a few reasons given for this non compliance. Some patients are simply not aware of the need for the medical treatment, or are in denial regarding their personal need for the treatment, regardless of how much emphasis a physician places on the importance of it. Other patients do not like types of treatment (such as dialysis) and try to avoid these types of procedures at all costs. Other patients are handicap or lack intelligence and therefore lack the necessary resources to obtain the treatment. Lastly, as stated above, some patients believe that God will treat them and they do not need medical intervention for their condition.

Sign of CKD-clinicians reported that most patients do not experience symptoms of CKD and thus, the disease progresses before it is diagnosed. Some patients report nausea and pain as a symptom of the disease but nephrologists agreed that these symptoms are typically not experienced until the disease has progressed to later stages.

CKD occurrence-all four nephrologists agreed that the majority of CKD patients are African American. Each nephrologist gave estimates that ranged from 70% to 95% of their patients that are African American. In addition, nephrologists agreed that typically the lower educated and less affluent community has a higher prevalence of CKD.

Transplant thoughts-nephrologists did not speak much about patient thoughts on kidney transplant. However, two nephrologists reported that they have had patients that believe that transplant is a ‘magical cure’ for CKD and if they have a transplant, their condition will immediately be improved and back to ‘normal’. Other nephrologists reported that some of their patients are very unsure of transplant, due to lack of information.

Home dialysis-a few of the nephrologists stated that for patients that meet requirements of home dialysis, they try to encourage this as a possible treatment option, simply because they see it as an easier and more comfortable type of treatment. The patient can do the dialysis in the comfort of their own home with a familiar support network, on their own schedule. However, these nephrologists declared that even when patients are eligible for this type of home treatment (meet a list of strict requirements), most are intimidated by the thought of doing the treatment at home and would much rather visit the dialysis clinic for the treatment.

Information availability-nephrologists also did not speak much about availability of information for patients but one did state that he believed that many patients do not have access to vital information regarding CKD, illustrating a need for more community and medical education.

### Nurses focus groups

#### Themes

Themes from the nurse clinicians’ files data are listed below (Table 2), along with the corresponding number of segments that each theme represented in the three files:

**Table 2:** Themes from nurses’ focus groups for African American patient perceptions on chronic kidney disease

**Reaction to chronic kidney disease (53 segments)**	**Definition of code**	**Number of occurrences**
Unsure of chronic kidney disease	Patient is unsure of disease	20
Denial reaction to chronic kidney disease	Patient is in denial after diagnosis	18
Accepting of chronic kidney disease	Patient is accepting of diagnosis	5
Patients don’t try to prevent	After diagnosis, patients admit they aren’t trying to prevent disease	3
All patients different in reaction	Patient reacts differently after diagnosis	3
Fear reaction to chronic kidney disease	Patient is afraid after diagnosis	2
Depression reaction to chronic kidney disease	Patient is in depression after diagnosis	1
Shock reaction to chronic kidney disease	Patient is in shock after diagnosis	1
**Thoughts on dialysis (44 segments)**	**Definition of code**	**Number of occurrences**
Fear of dialysis	Patient has fear of dialysis	13
Unsure of dialysis	Patient is unsure of dialsysis	8
Dialysis helps	Patient thinks dialysis helps treat chronic kidney disease	8
Patient does not like dialysis	Patient does not like dialysis	8
Shock of dialysis	Patient is in shock at dialysis treatment prescription	7
**Information availability (38 segments)**	**Definition of code**	**Number of occurrences**
Lack of information	Patient lacks information about disease	38
**Compliance to treatment (30 segments)**	**Definition of code**	**Number of occurrences**
Patient non-compliant to treatment	Patient is non compliant to prescribed treatment	25
Patient compliant to treatment	Patient is compliant to prescribed treatment	5
**Information source (28 segments)**	**Definition of code**	**Number of occurrences**
Information from experience	Patient got information from personal experience	15
Information from family	Patient got information from family	4
Information from physician	Patient got information from physician	4
Information from other patients	Patient got information from other patients with kidney disease	3
Information from Internet	Patient got information from Internet	2
**Transplant thoughts (27 segments)**	**Definition of code**	**Number of occurrences**
Unsure of transplant	Patient is unsure of transplant	12
Transplant risky	Patient thinks transplant is risky	10
Transplant helps	Patient thinks transplant helps kidney disease	5
**Reasons for non-compliance (26 segments)**	**Definition of code**	**Number of occurrences**
God will heal	Patient thinks God will heal and no need for medicinal treatment	12
Patient doesn’t want treatment	Patient doesn’t want treatment	6
Lack money for treatment	Patient lacks money for treatment	5
Medicine won’t work in Af Am population	Patient thinks medicine won’t work in African American population	3
**Thoughts on God (25 segments)**	**Definition of code**	**Number of occurrences**
God will heal	Patient thinks God will heal	12
God not responsible	Patient does not think God is responsible for kidney disease	7
Trust in God	Patient trusts God	6
**Types of treatment (22 segments)**	**Definition of code**	**Number of occurrences**
Dialysis is treatment for chronic kidney disease	Patient thinks dialysis is a treatment for chronic kidney disease	9
Transplant is a treatment	Patient thinks transplant is a treatment for chronic kidney disease	7
Home remedy is treatment	Patient thinks that home remedies can treat chronic kidney disease	5
Medication treatment for chronic kidney disease	Patient thinks medication is a treatment for chronic kidney disease	1
**Cause of chronic kidney disease (20 segments)**	**Definition of code**	**Number of occurrences**
Unsure of causes of chronic kidney disease	Patient is unsure of causes of chronic kidney disease	6
Blame others for chronic kidney disease	Patient blames others for chronic kidney disease	4
Stress causes chronic kidney disease	Patient thinks stress causes chronic kidney disease	3
Genetics causes chronic kidney disease	Patient thinks genetics causes chronic kidney disease	3
Blood pressure causes chronic kidney disease	Patient thinks high blood pressure causes chronic kidney disease	2
Diabetes causes chronic kidney disease	Patient thinks diabetes causes chronic kidney disease	2
**Reasons for compliance (12 segments)**	**Definition of code**	**Number of occurrences**
Socialization with treatment	Patient complies with treatment for socialization	8
Waiver for treatment	Patient gets a fee waiver for treatment	4
**Support network (9 segments)**	**Definition of code**	**Number of occurrences**
Lack support network	Patient lacks support	5
Good support network	Patient has good support	4
**Sign chronic kidney disease (7 segments)**	**Definition of code**	**Number of occurrences**
Swelling is sign of chronic kidney disease	Swelling is a symptom of chronic kidney disease	2
Tired is sign of chronic kidney disease	Being tired is a symptom of chronic kidney disease	2
No symptom for chronic kidney disease	Symptoms are hard to see in chronic kidney disease	2
Nausea is sign of chronic kidney disease	Nausea is a symptom of chronic kidney disease	1
**Home dialysis (6 segments)**	**Definition of code**	**Number of occurrences**
Home dialysis intimidating	Patient thinks home dialysis is intimidating	6
**Who urged treatment (1 segment)**	**Definition of code**	**Number of occurrences**
Family urges treatment	Family of patient urges treatment	1

Reaction to CKD (53), thoughts on dialysis (44), information availability (38), compliance to treatment (30), information source (28), transplant thoughts (27), reasons for non-compliance to treatment (26), thoughts on God (25), types of treatment (22), causes of CKD (20), reasons for compliance to treatment (12), support network (9), signs of CKD (7), home dialysis (6), who urged treatment (1).

During the focus groups, it was evident that the nurse clinicians were very aware of their patient’s knowledge, ideas, and beliefs surrounding CKD and its effects and consequences. The nurses were able to give detailed information on patient experiences with the condition. Some of the most common topics in the nursing focus groups centered around patient reactions to a diagnosis of CKD, patient thoughts on dialysis, availability of information to patients, patient compliance to treatment, information source for patients, patient thoughts on transplant, reasons for patient non-compliance to treatment, and thoughts on God.

### Most prevalent themes

#### Reaction to CKD

In the focus groups, all nurses spoke broadly and repeatedly about the uncertainty that their patients feel regarding CKD. Patients are typically unsure and confused about the effects of the disease and feel like they are lacking necessary education and information. Even if clinical staff members attempt to counsel and educate the patients, they are unsure if the patients are truly understanding and comprehending it. One nurse explained, “sometimes, I have had patients where you talk to them and, if you don’t really bring it down to a really basic level, they are not really sure exactly. And you can tell by the look on their faces, they are not truly comprehending what you are saying. You really have to simplify it and maybe sometimes that is not being done all the way, so they are coming in at partial understanding…”

It was also agreed upon in the interviews that all patients have vastly different reactions to CKD. The nurses established that there is a broad sense of denial in the kidney disease patient population. Patients are in denial regarding their condition and believe that either the physicians are incorrect in the diagnosis, or think that their condition will improve without medical treatment. Contrarily, a few nurses did report that some patients in their clinic are accepting of CKD. These patients typically have a family history of the disease or have some previous experience in dealing with the effects of the disease so are more accepting of the condition when they are diagnosed. These patients also realize that there are treatment options for CKD that can improve their overall health, and they are more likely to be compliant to the treatment protocols.

Lastly, a few nurses also described characteristic emotions of fear, depression, and shock as typical reactions to a diagnosis of CKD.

#### Thoughts on dialysis

All nurses spoke most commonly about the fear that patients feel when they are told they have to be put on dialysis. It was compared to an “alarm bell” that goes off for the patient and they become instantly fearful of the procedure and its effects. One nurse highlighted this concept by stating, “I’ve had patients say, you may as well go ahead and shoot me if I have to go on that machine", illustrating the intense emotions that correlate with the prospect of dialysis.

In addition, many patients are unsure about dialysis. They lack information and education about the dialysis process, including what it entails, what it does to help them, and the effects of it. This uncertainty, in turn increases their fear of the procedure. One nurse explained, “I don’t think chronic kidney disease is advertised really well in the African American community. Because I spoke with someone not very long ago and she was telling me that she had never met anyone that was on it… and most people, if they don’t know another patient that is on dialysis, they really don’t know a whole lot about what causes chronic kidney disease and what dialysis really is".

Nurses also explained that a lot of patients simply do not like the dialysis process. It is extremely time consuming, as patients have to visit the clinic a few times a week for an extended period of time. These visits disrupt daily life and activities and many patients feel that the entire process is a burden to their lifestyle and routine.

When patients are told that they are going to be put on dialysis, some exhibit emotions of shock and utter disbelief. As stated above, many patients are in denial about CKD so are in shock at the recommendation of dialysis. Sometimes patients can overcome this shock, reach acceptance of the necessary treatment steps, and follow through on treatment. However, if a patient does not accept the diagnosis of CKD and realize that treatment is vital, non compliance to treatment can occur.

Lastly, some patients do believe that dialysis will help them and is beneficial. These patients are typically in the minority and are the ones that remain compliant to treatment and take it very seriously. They believe that if they follow the instructions of their physicians, the dialysis will help them. In addition, these are the patients that admit that they do physically feel better after starting the dialysis treatment.

#### Information availability

In discussing the availability of information for CKD patients, all nurses in all focus group sessions agreed unanimously that patients are lacking adequate information regarding the effects of CKD and treatment options for the disease. The nurses believed that each patient needs to be allotted more time with their physician to allow for education. Nurses mentioned large events such as information sessions, support groups and rallies for diseases such as HIV and cancer; yet nothing similar or comparable exists for CKD, even though it can be just as widespread and serious of a disease. The dialysis center nurses discussed strict HIPAA laws that limit the advertising and information they can share. The center used to have newsletters and offer dialysis center tours for prospective patients but HIPAA laws now restrict both of these outlets of education and information sharing.

Nurses also recommended putting educational information sessions on the televisions in the dialysis center rooms. Patients sit in these dialysis rooms for many hours a day, a few times a week so there is a clear targeted audience. Putting short, captivating and memorable educational sessions on the televisions could give these patients exposure to vital health information.

#### Compliance to treatment

Almost all nurses in all focus groups agreed that CKD patients are typically not compliant to their prescribed treatment regimen. Many of these patients are in denial about their condition and do not want to seek necessary treatment. Other patients lack resources, such as funding, transportation, and support for the treatment. It was stated that a lot of the patients that refuse their critical treatment end up in the emergency room at their local hospital, time after time. Additional reasons for this non compliance to treatment regimens are given below.

However, supporting the idea already stated that some patients are different in their reactions to CKD, a few nurses admitted that they have had patients remain compliant with their prescribed treatments. These patients realize and understand the importance of the treatments in order to improve their condition and take their individual regimens very seriously. Unfortunately, the compliant patients are the minority.

#### Information source

The majority of CKD patients acquire their knowledge and information regarding the disease from personal experiences, mostly with other family members or friends with the disease. Unfortunately most of these experiences have been negative and lead to preconceived notions amongst these patients that CKD is a “horrid disease” and causes death. Patients also get information from their family members and physicians and some are able to find background information on the Internet and arrive at physician visits with lists of questions which helps facilitate information sharing from physicians.

The dialysis nurses specifically reported that they believe their patients get most of their information from other patients. Dialysis patients sit in the clinic a few times a week with other patients experiencing similar procedures, effects, and emotions. These patients share stories, insight and advice with each other. Dialysis nurses noted that this sharing of information in the dialysis clinic is not always beneficial because patients that have had a negative experience with a type of treatment want to share their experiences with everyone, which again causes negative preconceived notions among the patients.

#### Patient thoughts on transplant

When discussing patient thoughts on kidney transplant, nurses agreed that patients are typically unsure of this process. Patients do not have adequate information to make decisions about transplant and are uncertain whether they have the home support to help them prepare for and recover from surgery. In addition, the entire transplant medical process is lengthy so patients are not sure they can dedicate a sufficient amount of time to the process. The process requires multiple medical visits, tests and evaluations before the actual surgery can take place. One nurse in the Columbia focus group stated that the most ideal situation occurs in which a patient can get all the pre-testing and evaluations for a transplant completed in the hospital in one or two days. Otherwise, the patients spend a lot of time travelling to different appointments throughout a longer time period. The more appointments that need to be set, the higher likelihood of a patient forgetting and missing an appointment or not having transportation, which further delays the procedure. Also, some patients remain in denial about their diagnosis of CKD so cannot commit to a kidney transplant until they become accepting of their condition.

Patients also think that kidney transplant is risky so many choose not to pursue it. These patients have typically had a negative experience with a friend or family member that had a transplant that was not successful and thus have negative preconceived notions about the procedure. Other risks that patients are concerned about involve the spread of disease and secondary conditions that can result from a kidney transplant.

There were a few nurses from the different clinics that admitted that some patients do believe that kidney transplant can be beneficial for them. Most often these patients are very supportive of the transplant and dedicated to the treatment regimen because they are emotionally and physically drained from being on dialysis for an extended period of time.

#### Reasons for non-compliance to treatment

As stated above, the majority of the CKD population is generally not compliant to their prescribed treatment regime. The focus group nurses gave a few different reasons for this non compliance, with different reasoning coming from the different locations.

The nurses in the Columbia, South Carolina medical center reported that a lot of their patients do not want to adhere to their prescribed treatment because they believe God will heal them and medical treatment is not necessary. They conveyed that patients broadcast their faith and religious views and exclaim that prayer is all that they need to heal and recover from CKD. As one nurse said, “…some of them probably feel like, if they do accept any medical care then that’s showing a lack of faith".

The nurses from MUSC in Charleston, South Carolina reported that some patients simply do not want the treatment and thus do not comply with it. As stated above, many of the treatment protocols are very time consuming and disruptive to daily life, and also cause negative side effects which patients want to avoid. MUSC nurses also relayed the idea that many of their patients cannot afford CKD treatment and simply do not have the funds to pay for the needed medical care.

Lastly, some of the nurses in the dialysis clinic stated that their patients have expressed the belief that some of the treatment medications do not work in the African American population. African American patients that have heard this idea do not feel the need to adhere to their prescribed treatment because they do not believe it will work. The nurses had mixed feelings about this concept and whether it is entirely true but believed it to be a reason for treatment non-compliance.

#### Thoughts on God

The nurses from all three focus groups discussed the role of patient faith and religion in different aspects of CKD. As stated above, the Columbia, South Carolina nurses reported that a lot of their patients do not comply with prescribed medical treatment because they believe that God will heal them and medical intervention is unnecessary. All nursing focus groups also reported that patients do not think that God is responsible for their CKD. Patients do not think that God is trying to punish them and a lot of patients trust that God will look over their situation and keep them safe.

### Other facets discussed in the focus groups

In addition to the main themes discussed above, nurses volunteered other opinions and experiences of their patients, relating to CKD.

Types of treatment-nurses reported that patients are knowledgeable about types of treatment available for CKD. The majority of patients with the condition are aware dialysis and kidney transplant are the main types of treatment for the disease. The MUSC nurses in Charleston, South Carolina patients also conveyed that their patients occasionally mention other types of “home remedies” for CKD treatment, which can include unique herbs and non-FDA approved medications.

Causes of CKD-all three focus groups of nurses reported consistently that most patients are unsure of the causes of CKD. It was repeatedly stated in all interviews that patients do not have enough information readily available to them and are not educated enough by their physicians and other clinical staff advisors. The Columbia, South Carolina nurses also confessed that they have heard their patients blame others for their CKD. The patients claim that they did not have the disease until they started going to the clinical visits and someone in the clinic or hospital was the cause of this disease. Lastly, some patients do realize that high blood pressure, diabetes and genetics can play a role in kidney disease, although these patients are typically in the minority.

Reasons for compliance to treatment- Although as stated above that the majority of patients are not compliant with prescribed treatment regimens for CKD, there are a handful of patients that do remain compliant. Nurses gave two main reasons for this compliance, with the main factor being the socialization involved in treatment, specifically dialysis. Patients in dialysis visit the same medical center and see the same clinicians at least a few times a week for a long time period. At the medical center, they typically sit in a room for extended intervals of time with other dialysis patients so are able to form strong supporting bonds with other patients and the clinical staff. In the interviews, a few nurses referred to the patients in their dialysis clinic as a small ‘family’ because they were so close knit. In addition, some patients are able to receive governmental financial waivers for their CKD treatment so they are not fully responsible for the charges incurred. These waivers increase the odds of treatment compliance for some under privileged patients.

Support network-In speaking about the support network available for most patients, the nurses affirmed that each patient has different levels of support. Some patients have a secure and stable support network of family and friends while other patients are completely lacking any type of support. A few nurses stated that a lot of patients are able to seek support not only from family and friends but also from their church congregation and minister. The patients that lack a support network typically have a more difficult time adhering to their prescribed treatment regimen and being present for all medical appointments.

Sign of CKD-Nurses related a few different signs and symptoms that cause patients to seek medical attention for CKD, including swelling, fatigue, and nausea. However, some patients do not report any symptoms of the condition. These patients with no noticeable symptoms generally are not as compliant with treatment regimens because they are in denial and believe that they are well and not in need of treatment. Medical staff must work to convince these patients that they do need the treatment in order to fight the disease.

Home dialysis-Only the dialysis center nurses spoke about home dialysis in the focus group interviews. These nurses all unanimously agreed that patients find the prospect of home dialysis very intimidating. There were many different reasons given as to why patients feel intimidated, including their personal lack of confidence and lack of education in administering dialysis. Even though the dialysis center staff offers to train them to be able to administer their own dialysis treatment at home, many patients would prefer someone else (the medical staff) do the treatment for them. Also, some patients lack the needed home support to go through the dialysis process and others simply enjoy the socialization that occurs when going to the dialysis center.

## Discussion

The data from the four interview sessions found that nephrologists were able to discuss multiple facets of their patient’s personal experiences and opinions relating to CKD. It was reported that patients have a wide range of reactions after a diagnosis of CKD and explained that this condition is very difficult to understand. Patients are somewhat knowledgeable about types of treatments available and realize that they should undergo changes in their lifestyle and that their typical daily routine will be altered, after a diagnosis of CKD. However, nephrologists admitted that although some patients are aware of these concepts, other patients still lack the knowledge and education of the importance of medical treatment and lifestyle changes for CKD. In addition, even among the patients who do understand the importance of treatment, many try to avoid medical procedures and will refuse necessary medical care. They are non-compliant with the prescribed treatment regimen because they are fearful of it and simply do not like undergoing medical treatment. In the interview sessions, physicians admitted that patients are able to discuss a few possible causes of CKD but the majority of patients remain unsure of the causes.

Nephrologists also spoke of racial and demographic differences in CKD prevalence. It was agreed upon that African Americans, as well as lower educated and less affluent individuals have a higher incidence of the disease. Also, African Americans seem to have more personal experience with CKD, as they have known more friends or family members with the condition and experienced first -hand the effects of the disease. African Americans typically trust that God will take care of them and depend on their faith and spirituality to guide them through an illness or health related condition.

It was evident in the focus groups that nurses from the three different locations were very knowledgeable about their patient’s experiences, thoughts, and opinions on CKD. Nurses spoke very openly about the patients reactions to CKD and reported that the most common reactions to a diagnosis of CKD are uncertainty and denial. Patients are also typically unsure of the causes of CKD and exhibit uncertainty and fear when discussing dialysis and kidney transplant. The majority of patients are not compliant with their prescribed treatment regimen, mostly due to this lack of knowledge and available information, as well as feelings of denial. Patients that do have knowledge of CKD and its treatments typically receive it from personal experiences with family members, friends, or other CKD patients which may not always be beneficial because it can cause negative preconceived notions in the patient before treatment even begins. The majority of patients exhibit high levels of faith and spirituality and depend on God to guide them through the disease process.

This study does have limitations. First, the sample participants volunteered for the study and therefore might not represent the entire population. Second, the study includes perceptions from only a small number of participants and again may not be fully representative.

## Conclusion

Although the nurses and physicians had varying perspectives, they also shared similar viewpoints related to the patients’ perception of their disease. According to both types of clinicians, patients are clearly lacking valuable and vital knowledge of CKD, causes of the disease, types of available treatments, and the benefits of these treatments.

Both the nephrologists and nurse clinicians cited a fear of dialysis and a lack of information as common issues for African American patients with CKD. While the participants of this study did not comment on how to overcome these challenges, possible strategies might include diabetes case managers and educators. It is possible, too, that the fear of dialysis and the lack of information may be related. It is possible that the fear exists because factual information about dialysis and CKD are not readily available. Fear is also generally associated with low self-efficacy, and patient centered education could lead to better self-management.

This information can be used in the medical field in numerous ways. The findings from these focus groups and the interviews with nephrologists are in agreement with and enhance the findings of studies performed and reported previously. Patients need to be better educated on the causes and treatments available for CKD and instructed that medical treatment is essential for survival in life with CKD. Education is vital to prevention and treatment of CKD and without education, CKD incidence will only worsen. In order to improve knowledge, nurses recommended more education from physicians (longer clinic appointment time slots), educational videos in the dialysis center rooms, and community programs and support groups. The educational programs should focus on the cultures, customs, and spirituality levels of all high risk populations, in order to reach the highest level of patient needs. Since CKD is most prevalent in the African American, lower educated, and less affluent communities, education needs to be focused in these areas, with special attention paid to focusing on these individual customs, cultures, and spirituality.

Additionally, since many patients are not compliant with prescribed treatments for their condition, support networks could be made available to ensure patients realize the importance of their individualized medical care and also make certain that they follow through on the prescribed treatments and lifestyle changes. Since the nurses in the focus groups repeatedly stated that each patient is different in their lifestyle, personal reactions, and emotions, educational opportunities need to be varied and widespread in order to reach the largest possible population.

## Abbreviation

CKD: Chronic kidney disease.

## Competing interests

The authors do not have any competing interests to report.

## Authors’ contributions

AK participated in the interpretation of data, writing of the manuscript, and editing of the manuscript. EJ participated in the analysis of data and writing of the manuscript. KS participated in the data collection and analysis. KC participated in the overall supervision and study design and the editing of the manuscript. PB provided supervision, conceptual design, and manuscript preparation. All authors read and approved the final manuscript.

## Authors’ information

AK, PhD is an Associate Professor, Department of Health Care Leadership and Management at MUSC. EJ, MHA is a doctoral student in the Department of Health Sciences and Research at MUSC. KS, DPH, is a Professor, Department of Health Care Leadership and Management, MUSC. KC, MD, PhD, is a Professor of Surgery, Division of Transplantion, MUSC. PB, MD, is Professor of Surgery and Chair of Transplantation at MUSC.

## Pre-publication history

The pre-publication history for this paper can be accessed here:

http://www.biomedcentral.com/1471-2369/15/112/prepub

## References

[B1] Centers for Disease Control and Prevention National Chronic Kidney Disease Fact Sheet: general information and national estimates on chronic kidney disease in the United States, 2010 2010 Atlanta, GA: U.S. Department of Health and Human Services, CDC

[B2] National Kidney Foundation Kidney Disease Statistics for the United States http://kidney.niddk.nih.gov/kudiseases/pubs/kustats/#3. Accessed 11/6/13

[B3] Agodoa L African American Study of Kidney Disease and hypertension (AASK)--clinical trial update Ethn Dis 1998 8 2 249 253 9681290

[B4] Bomback AS Kshirsagar AV Whaley-Connell AT Chen SC Li S Klemmer PJ McCullough PA Racial differences in kidney function among individuals with obesity and metabolic syndrome: results from the Kidney Early Evaluation Program (KEEP) Am J Kidney Dis 2010 55 3 S4 S14 10.1053/j.ajkd.2009.10.045 20172446

[B5] Bruce MA Beech BM Crook ED Sims M Wyatt SB Flessner MF Taylor HA Williams DR Akylbekova EL Ikizler TA Association of socioeconomic status and CKD among African Americans: the Jackson Heart Study Am J Kidney Dis 2010 55 6 1001 1008 10.1053/j.ajkd.2010.01.016 20381223 PMC2876216

[B6] Crews DC Charles RF Evans MK Zonderman AB Powe NR Poverty, race, and CKD in a racially and socioeconomically diverse urban population Am J Kidney Dis 2010 55 6 992 1000 10.1053/j.ajkd.2009.12.032 20207457 PMC2876201

[B7] Brown WW Peters RM Ohmit SE Keane WF Collins A Chen SC King K Klag MJ Molony DA Flack JM Early detection of kidney disease in community settings: the Kidney Early Evaluation Program (KEEP) Am J Kidney Dis 2003 42 1 22 35 10.1016/S0272-6386(03)00405-0 12830453

[B8] Lea JP Nicholas SB Diabetes mellitus and hypertension: key risk factors for kidney disease J Natl Med Assoc 2002 94 8 Suppl 7S 15S 12152917 PMC2594170

[B9] Nzerue CM Demissochew H Tucker JK Race and kidney disease: role of social and environmental factors J Natl Med Assoc 2002 94 8 Suppl 28S 38S 12152910 PMC2594163

[B10] Lenz O Durga P Mekala D Fornoni A Metz D Roth D Barriers to successful care for chronic kidney disease BMC Nephrol 2005 6 11 10.1186/1471-2369-6-11 16250919 PMC1283975

[B11] Tanyi R Werner J Spirituality in African American and Caucasian women with end-stage Renal Diseaseon Hemodialysis treatment Health Care Women Int 2007 28 141 154 10.1080/07399330601128486 17364977

[B12] Jennette CE Vupputuri S Hogan SL Shoham DA Falk RJ Harward DH Community perspectives on kidney disease and health promotion from at-risk populations in rural North Carolina, USA Rural Remote Health 2010 10 2 1388 20504048

[B13] Waterman AD Browne T Waterman BM Gladstone EH Hostetter T Attitudes and behaviors of African Americans regarding early detection of kidney disease Am J Kidney Dis 2008 51 4 554 562 10.1053/j.ajkd.2007.12.020 18371531

[B14] Tan AU Hoffman B Rosas SE Patient perception of risk factors associated with chronic kidney disease morbidity and mortality Ethn Dis 2010 20 2 106 110 20503888

[B15] Boulware LE Carson KA Troll MU Powe NR Cooper LA Perceived susceptibility to chronic kidney disease among high-risk patients seen in primary care practices J Gen Intern Med 2009 24 10 1123 1129 10.1007/s11606-009-1086-6 19711135 PMC2762510

[B16] Jean-Baptiste R Psychosocial factors affecting end stage renal disease patient compliance with hemodialysis attendance Dissertation Abstracts International: Section B: The Sciences and Engineering 2002 62 12-B 6024

[B17] Hyden LC Bulow PH Who’s talking-drawing conclusions from focus groups-some methodological considerations Int J Soc Res Methodol 2003 6 4 305 321 10.1080/13645570210124865

[B18] Shi L Health services research methods 2008 2 New York: Delmar Cengage Learning

[B19] Morgan D Focus groups Annu Rev Sociol 1996 22 129 152 10.1146/annurev.soc.22.1.129

[B20] Bradley E Curry L Devers K Qualitative data analysis for health services research: developing taxonomy, themes and theory Health Research and Educational Trust 2006 10 1758 1772 10.1111/j.1475-6773.2006.00684.x PMC1955280 17286625

[B21] Glaser BG Emergence V forcing basics of grounded theory analysis 1992 Mill Valley, CA: Sociology Press

[B22] Glaser BG Strauss AL The discovery of grounded research: strategies for qualitative research 1967 New York: Aldine De Gruyter

[B23] Patton M Qualitative research and evaluation methods 2002 3d Thousand Oaks, CA: Sage Publications

